# 18 Does overweight enhance foot disorders in patients with juvenile idiopathic arthritis?

**DOI:** 10.1093/rheumatology/keac496.014

**Published:** 2022-10-07

**Authors:** H. Ferjani Dghaies, M Moalla, D Ben Nessib, W Triki, K Maatallah, Dh Kaffel, W Hamdi

**Affiliations:** Kassab Institute of Orthopaedics, Tunisia

## Abstract

**Background:**

Juvenile idiopathic arthritis (JIA) is chronic inflammatory arthritis of childhood that may result in persistent and disabling foot impairments. Foot disorders are common in JIA with a prevalence estimated at over 90%. Many factors can worsen foot disorders in these patients. The influence of weight on foot disorders is poorly studied.

**Objectives:**

To examine associations between weight and foot disorders in patients with JIA.

**Methods:**

Patients with a diagnosis of JIA, based on the International League of Associations for Rheumatology (ILAR) criteria were included. Age, gender, weight, characteristics of the disease and of foot pain were noted. A specialized podiatric examination was performed.

**Results:**

Thirty-two patients were included. The mean age was 12.2 ± 2.9 [5–18]. Forty-three percent of the patients were boys (*n* = 14). The mean age of disease onset was 8.5 ± 3.9 [3–15]. Only one patient had a triggering factor (elbow fracture). The most common type of JIA was oligoarthritis in 12 cases, then enthesitis-related in 8 cases, polyarthritis without rheumatoid factor in 3 cases, polyarthritis with positive rheumatoid factor in 1 case, psoriatic arthritis in 3 cases, systemic arthritis in 1 case and undifferentiated arthritis in 4 cases.

The mean weight was 43.5 kg [17–98]. Only 28% (*n* = 9) of the children had a normal weight for their age, 41% (*n* = 13) of them had a low weight for their age and 31% (*n* = 10) of them had overweight. Ten patients had foot pain: hindfoot pain in 5 patients, midfoot pain in 3 patients, and forefoot pain in 2 patients. Foot deformities were found in 78.1% of the patients (*n* = 27): flat foot in 39% (*n* = 13) of the patients and pes cavus in 39% (*n* = 13).

There was no association between overweight and foot pain (*p* = 0,14) or hindfoot pain (*p* = 0.08). However, overweight was associated with a forefoot (*p* = 0.01) and midfoot (*p* = 0.01) pain. Overweight was not associated with foot deformities (*p* = 0.1).

**Conclusion:**

This study showed that weight is associated with foot pain especially forefoot and midfoot pain. A healthy lifestyle and a normal weight are fundamental to preventing foot pain at an early age in patients with JIA and improving their quality of life.

